# The prognostic importance of glioblastoma size and shape

**DOI:** 10.1007/s00701-024-06351-0

**Published:** 2024-11-12

**Authors:** Claes Johnstad, Ingerid Reinertsen, Erik Thurin, Tora Dunås, David Bouget, Lisa M Sagberg, Asgeir S Jakola, Ole Solheim

**Affiliations:** 1 Department of Neuromedicine and Movement Science, Faculty of Medicine and Health Sciences, Norwegian University of Science and Technology, Trondheim, Norway; 2 Department of Health Research, SINTEF Digital, Trondheim, Norway; 3 Department of Circulation and Medical Imaging, Faculty of Medicine and Health Sciences, Norwegian University of Science and Technology, Trondheim, Norway; 4 Department of Radiology, Sahlgrenska University Hospital, Gothenburg, Sweden; 5 Department of Clinical Neuroscience, Institute of Neuroscience and Physiology, Sahlgrenska Academy, University of Gothenburg, Gothenburg, Sweden; 6 Department of Neurosurgery, St. Olav’s Hospital, Trondheim University Hospital, Trondheim, Norway; 7 Department of Public Health and Nursing, Faculty of Medicine and Health Sciences, Norwegian University of Science and Technology, Trondheim, Norway; 8 Department of Neurosurgery, Sahlgrenska University Hospital, Gothenburg, Sweden; 9 Department of Neuromedicine and Movement Science, Norwegian University of Science and Technology, Trondheim, Norway

**Keywords:** Glioblastoma, MRI, Overall survival, Tumor shape, Tumor size

## Abstract

**Purpose:**

Extent of resection, MGMT promoter methylation status, age, functional level, and residual tumor volume are established prognostic factors for overall survival in glioblastoma patients. Preoperative tumor volume has also been investigated, but the results have been inconclusive. We hypothesized that the surface area and the shape were more representative of the tumor’s infiltrative capacities, and thus, the purpose of this study was to assess the prognostic value of tumor size and shape in patients with glioblastoma.

**Methods:**

In total, 271 patients with primary, unifocal glioblastoma were included from two centers in Norway and Sweden, respectively. All tumors were automatically segmented on preoperative MRI scans and manually validated. Tumor volume was used as a measurement of size, whereas sphericity index and area-to-volume ratio defined the shape complexity of the tumor. Contact surface area of the tumor was considered a measurement of both size and shape. Multivariable Cox proportional hazards models were used to assess the prognostic value of the respective tumor measurements, with previously established prognostic factors as covariates.

**Results:**

There were no associations between preoperative tumor volume and overall survival. Contact surface area (HR = 1.013, *p* = 0.002) and sphericity index (HR = 2.223, *p* = 0.001) were both significant independent prognostic factors for survival in the multivariable Cox models. Contact surface area was also associated with MGMT promoter methylation (*p* = 0.039) and extent of resection (*p* = 0.017).

**Conclusion:**

Tumor shape complexity appears to be an independent prognostic factor in glioblastoma patients and may also be associated with MGMT promoter methylation status and extent of surgical resection.

## Background

Glioblastomas usually appear as contrast-enhancing lesions with a hypointense central necrosis on T1-weighted MRI (magnetic resonance imaging). Despite maximal safe tumor resection with adjuvant radiochemotherapy, glioblastoma patients have a median survival of approximately 15 months [11]. Prognostic factors like MGMT (O^6^-methylguanine-DNA methyltransferase) promoter methylation status, extent of resection (EOR), residual tumor volume, functional level, and age, are well established. On the other hand, factors including the importance of pretreatment tumor volume are still debated. Preoperative tumor volume has been linked with survival in some [2, 10, 17], but not all studies [3, 14]. However, as the infiltration happens at the margins of the tumor, tumor shape and surface area may be more representative of the tumor’s infiltrative capacities than tumor volume. Previous studies have found that tumor surface area may be a stronger prognostic factor than tumor volume in glioblastoma patients [5, 16, 21]. Tumor shape has also been reported as a significant prognostic factor in one article [21] but was deemed insignificant in another [16]. In the present study, volumes, tumor shapes, and surface areas were computed based on volume segmentations of the glioblastomas. We explored the potential prognostic value of four different measurements of tumor size or shape and assessed the association to other known prognostic factors.

## Methods and materials

### Data

The sources of data for this project were Sahlgrenska University Hospital (Gothenburg, Sweden) and the Central Norway Brain Tumor registry and biobank, a population-based registry initiated in 2015 by St. Olav’s University Hospital (Trondheim, Norway). From these two centers, data from respectively 189 and 140 primary glioblastoma resections in adults from 2012 to 2022 were included, and in total 271 were analyzed. Simple biopsies were not included in this study. Patients with missing MRI scans or with multifocal tumors, defined as tumor segments with a gap of one or more voxels on postoperative MRI, were excluded. Pre- and postoperative MRI scans were taken within 72 h of surgery. The glioblastoma diagnoses were initially based on the World Health Organization (WHO) classification system relevant at the time of surgery (2007, 2016 or 2021) but later reclassified according to the WHO 2021 classification based on available IDH (isocitrate dehydrogenase) mutation status (available for *n* = 268 [99%]), excluding all IDH-mutant astrocytomas.

#### Tumor size measurements

**Fig. 1:**
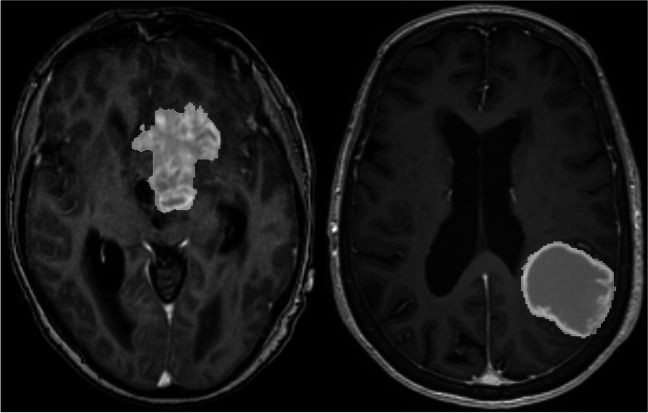
Example of a segmentation of an irregularly shaped tumor on the left and a more spherical tumor on the right

The glioblastomas were automatically segmented from preoperative, post-gadolinium T1-weighted MRI scans using a deep learning-based method developed by our research group [4], and the outputs were manually validated and, if needed, adjusted by a trained medical student. Figure 1 shows an example of two segmentations showing a regular and an irregular tumor, respectively. Total tumor surface areas (TSA) and tumor volumes were computed in 3DSlicer 5.4.0 using the “Labelmap statistics” tool [19]. To estimate the contact surface area (CSA) between the tumor and brain parenchyma, tumor surface areas adjacent to the dura were manually segmented and subtracted from the TSA. The sphericity index (SI) was calculated as the quotient of the TSA and the surface area of a sphere with equal volume as the tumor. The area-to-volume ratio (A/V) was defined as the ratio between the TSA and the volume of the tumor. Postoperative tumor volumes and EOR were automatically assessed using Raidionics [13].

#### Other measurements

For the Kaplan-Meier curves, gross total resection (GTR) and subtotal resection (STR) were defined as EOR of 100% and less than 100%, respectively. Otherwise, EOR was analyzed as a continuous variable. Postoperative KPS was estimated at one month postoperatively. Overall survival was defined as days from time of diagnosis until either death or end of follow-up on December 31, 2022.

### Statistical analyses

Statistical analyses were conducted in RStudio, version 2023.06.0 + 421. Density curves and Shapiro-Wilk test showed non-normal distribution of the data. Thus, we used Mann-Whitney U test for binary and Kruskal-Wallis test for polytomous variables. Correlations between continuous variables were assessed with Spearman’s correlation coefficient. Overall survival was investigated using univariable and multivariable Cox proportional hazards regression models and Kaplan-Meier curves. The covariates of the multivariable models were based on the results of the univariate models. Postoperative Karnofsky performance status (KPS) naturally loses its prognostic value with more time passed from its assessment, thus substantially violating the proportionality assumption of the Cox model. Consequently, postoperative KPS was stratified using the step function, resulting in one hazard ratio (HR) for KPS before 300 days and one HR for KPS after 300 days postoperatively [22]. Although such stratification may reduce power and external validity of the model, satisfying the proportionality assumption was deemed more important for the reliability of the estimated hazard ratios in the present study. Harrell’s concordance index (C-index) and Akaike’s information criterion (AIC) were used to compare the multivariable Cox models [1, 12]. P-values less than 0.05 were considered statistically significant.

### Ethics

The project was approved by the Regional Committee for Medical and Health Research Ethics (REK) in Norway (REK-reference 2019/510) and the Swedish Ethical Review Authority (Dnr: 702 − 18). The study was conducted in accordance with the Declaration of Helsinki, and all patients provided either written informed consent (Norway) or passive consent (Sweden).

## Results

**Fig. 2:**
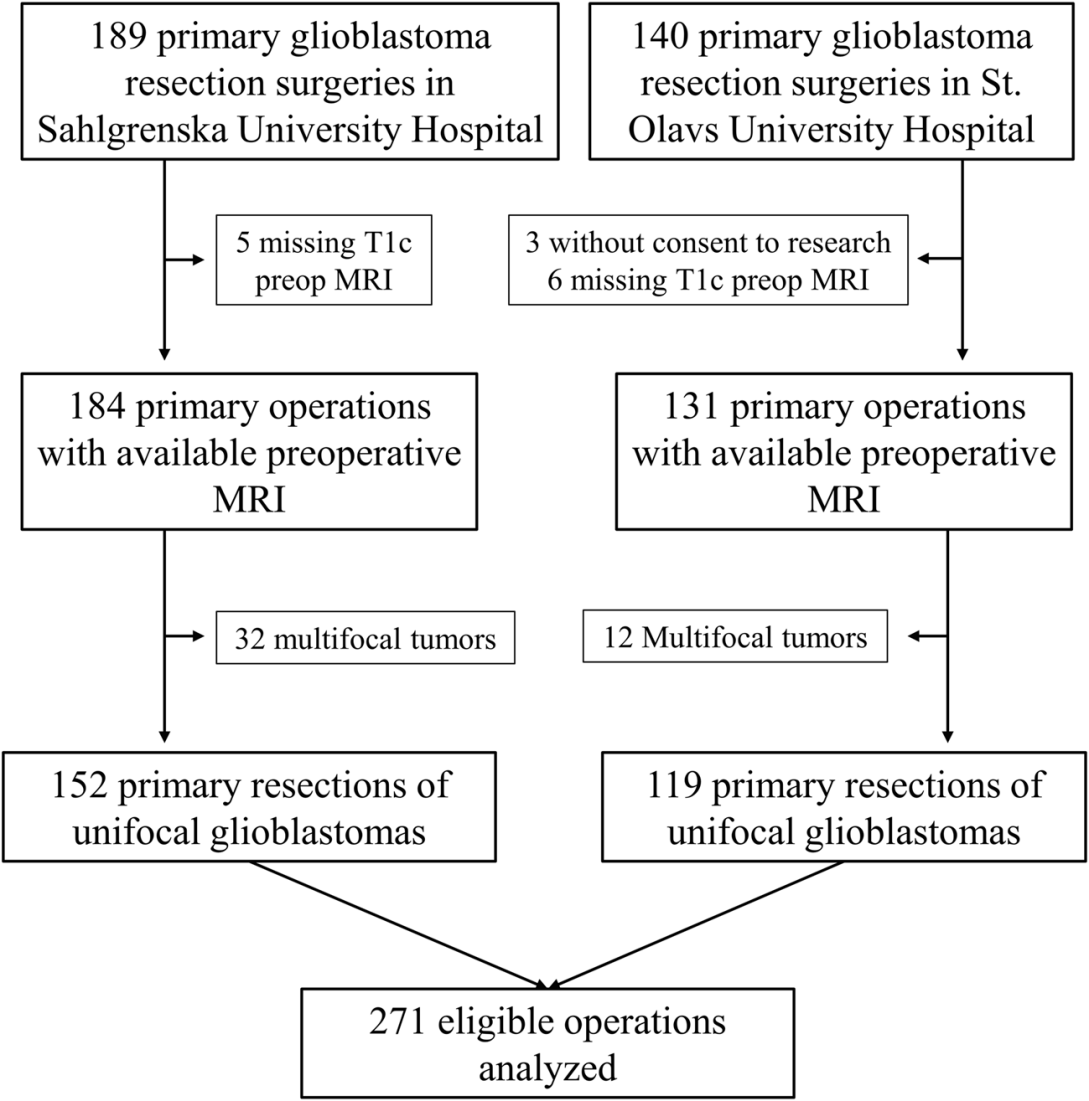
Flowchart of the inclusion process. MRI: magnetic resonance imaging; T1c: post-gadolinium T1-weighted

 Figure 2 shows the inclusion process. In the respective centers, 189 and 140 primary glioblastoma resections were performed. After excluding operations with missing post-gadolinium T1-weighted MRI scans or segmentations, multifocal tumors, and patients who did not consent to research, we analyzed in total 271 eligible operations.

Table 1 shows the median with first and third quartile of the respective variables of interest. Two hundred thirty-five (87%) of the glioblastomas were located adjacent to the dura mater, which covered a median 7.5% of the surface area.

**Table 1:** Descriptive statistics

Variable	Median	Q_1_-Q_3_
TSA (cm^2^)	61.4	30.2–96.3
CSA (cm^2^)	56.9	27.1–87.4
Volume (mL)	30.7	10.1–56.0
A/V (cm^−1^)	1.98	1.51–2.65
SI	1.26	1.16–1.49
EOR	98.4%	92.5-100.0%
Survival (months)	13.9	9.2–20.4

Table 1 Median and interquartile range of tumor measures and survival. *TSA* tumor surface area, *CSA* contact surface area, *A/V* area-volume-ratio, *SI* sphericity index, *EOR* extent of resection

**Fig. 3:**
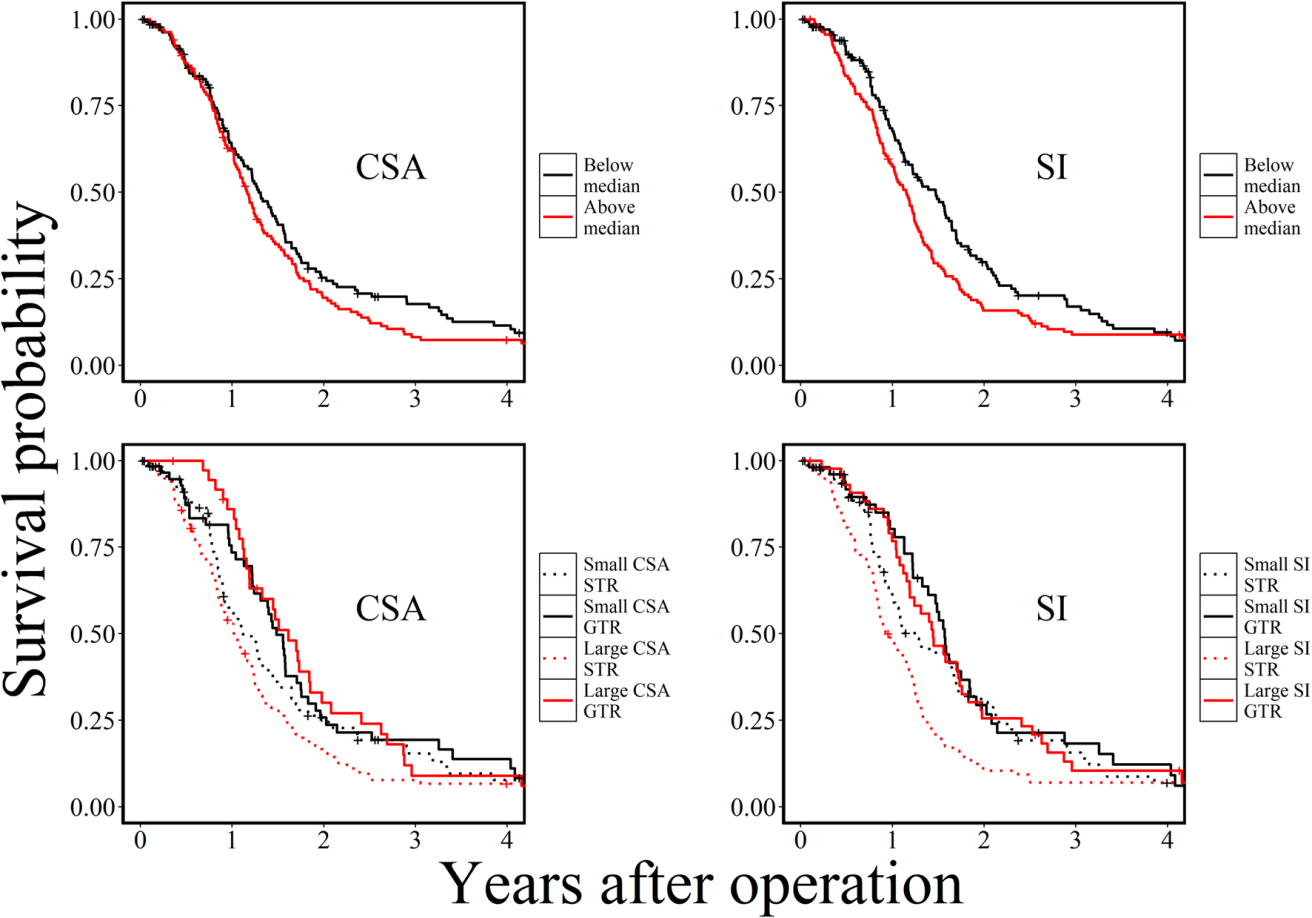
Kaplan-Meier curves for tumors with CSA and SI below and above median, respectively (top row) and Kaplan-Meier curves for CSA and SI in tumors with and without gross total resection, respectively (bottom row). CSA: contact surface area; SI: sphericity index; STR: subtotal resection; GTR: gross total resection

 Table 2 shows a summary of the univariable statistical analyses. There was an inverse Spearman correlation between CSA and EOR (r_s_ = -0.147, *p* = 0.017). The Mann-Whitney U test showed a positive association between greater CSA and MGMT promoter methylation (*p* = 0.039). The univariable Cox models found that CSA and SI were significantly associated with survival, but preoperative tumor volume and A/V were not. No other significant associations were observed in the univariable analyses. To illustrate the relationship between tumor shape and EOR, Fig. 3 shows Kaplan-Meier curves of CSA and SI by value and whether the patient underwent gross total or subtotal resection.

**Table 2:** Univariable analyses

	CSA		Volume		A/V		SI	
Spearman Correlation Coefficient								
	r_s_	p	r_s_	p	r_s_	p	r_s_	p
Age (*n* = 271)	-0.097	0.111	-0.100	0.102	0.060	0.328	-0.079	0.193
EOR (*n* = 264)	-0.147	**0.017**	-0.117	0.057	0.045	0.471	-0.099	0.110
KPS (*n* = 233)	-0.091	0.167	-0.092	0.163	0.064	0.329	-0.012	0.851
Mann-Whitney U test								
	Median cm^2^	p	Median mL	p	Median cm^−1^	p	Median	P
Sex		0.575		0.976		0.456		0.388
Female (*n* = 108)	55.7		32.4		1.90		1.26	
Male (*n* = 163)	57.1		30.4		1.98		1.27	
MGMT		**0.039**		0.077		0.801		0.237
Methylated (*n* = 122)	60.2		35.6		2.01		1.27	
Active (*n* = 143)	53.3		27.8		1.93		1.25	
Univariable Cox Proportional Hazards Model								
	HR	p	HR	p	HR	p	HR	p
Survival (*n* = 271)	1.003	**0.039**	1.002	0.349	0.971	0.630	1.645	**0.031**

Table 2 Summary of statistical analyses. *CSA* contact surface area, *A/V* area-volume-ratio, *SI* sphericity index, *rs* spearman correlation coefficient, *EOR* Extent of resection, *KPS* Karnofsky performance status, *MGMT* O-6-methylguanine-DNA methyltransferase, *HR* hazard ratio

The results of the two multivariable Cox models, with previously established prognostic factors and the respective tumor measurements CSA and SI as predictors, are presented in Table 3. CSA (*p* = 0.0022) and SI (*p* = 0.0015) both remained significantly associated with overall survival when adjusting for preoperative tumor volume, MGMT methylation status, age, EOR, and postoperative KPS. The AIC of the two models were equal, and the C-indices were 0.748 and 0.743, respectively.

**Table 3:** Multivariable Cox models

Predictors		HR	HR 95% CI	*p*
CSA model (C-index = 0.748, AIC = 1624) [*n* = 194]				
CSA		1.013	1.005–1.022	**0.0022**
Preoperative volume		0.988	0.976-1.000	0.057
Methylated MGMT promoter		0.463	0.342–0.628	**< 0.0001**
Age		1.038	1.023–1.052	**< 0.0001**
Postoperative KPS	< 300 days	0.935	0.920–0.951	**< 0.0001**
	> 300 days	0.990	0.976–1.005	0.18
EOR		0.161	0.039–0.665	**0.012**
SI model (C-index = 0.743, AIC = 1624) [*n* = 194]				
SI		2.223	1.360–3.660	**0.0015**
Preoperative volume		1.004	0.998–1.009	0.19
Methylated MGMT promoter		0.475	0.352–0.641	**< 0.0001**
Age		1.037	1.023–1.052	**< 0.0001**
Postoperative KPS	< 300 days	0.934	0.919–0.950	**< 0.0001**
	> 300 days	0.989	0.975–1.004	0.15
EOR		0.142	0.035–0.574	**0.0062**

Table 3 Results of the multivariable Cox proportional hazards models. *HR* hazard ratio, *CSA* contact surface area, *AIC* Akaike's information criterion, *C-index* Harrell's concordance index, *MGMT* O-6-methylguanine-DNA methyltransferase, *EOR* extent of resection, *KPS* Karnofsky performance status

## Discussion

In this two-center retrospective cohort study, we assessed the prognostic value of tumor size and shape in patients with primary, unifocal glioblastoma. Survival was not significantly associated with preoperative tumor volume, but with tumor shape, assessed as SI and CSA. While irregularly shaped tumors are more often MGMT promoter methylated, tumors with a complex shape and large surface area in contact with brain tissue appear to independently predict shorter survival and lower EOR.

Tumor volume is one of the more investigated potential prognostic factors but with inconclusive results in the literature, as some studies showed significant prognostic value [7, 10, 17], while others reported no prognostic value [3, 14]. In our analyses, we found no association between pretreatment tumor volume and overall survival. Nevertheless, extents of resection and the postoperative tumor volume were found to be prognostic factors in numerous studies [10, 15, 17]. The potential importance of tumor surface area is far less studied but has previously been assessed in glioblastoma [5, 16, 21], whereas contact surface area has not been explored in the current literature. Like the present study, one of the aforementioned studies also found associations between the sphericity of the tumors and survival [21]. Building on the hypotheses of these studies, we explored the potential prognostic value of size and shape.

In the univariable Cox models, CSA and SI were both significant prognostic factors, while A/V and volume were insignificant, in accordance with a previous radiomics study [21]. The results of the univariate analyses should be interpreted with potential confounders like age or MGMT promoter methylation status in mind. With the currently established prognostic factors of glioblastoma as covariates, the multivariable Cox models showed that SI and CSA were still significant predictor variables, thus supporting the independent prognostic value of tumor shape. Although pretreatment tumor volume was not associated with overall survival in the univariable analyses, we included it as a covariate in the multivariable models to adjust for any potential interactions with tumor shape and EOR. Consistent with the univariable analyses, the previously established prognostic factors were also significantly associated with overall survival in the multivariable models, whereas preoperative tumor volume remained an insignificant prognostic factor. Both models had an AIC of 1624, and the respective C-indices were 0.748 and 0.743, suggesting that there is no significant difference in prediction of overall survival between the two models.

Based on our data, it seems that tumors with a greater surface area in contact with brain parenchyma and tumors with a more complex shape predict a worse prognosis for glioblastoma patients. Whereas SI is a measurement of tumor shape alone, CSA was interpreted as a reflection of both the size and the shape complexity of the tumor, in accordance with previous research [5]. We found no association between tumor volume and overall survival, indicating that shape is of more importance than tumor size. One explanation could be that tumors with irregular surfaces perhaps have greater infiltrative potential. Another hypothesis is that tumors adjacent to larger white matter tracts or vasculature will typically infiltrate along these paths [6, 8], perhaps contributing both to the irregular shape of the tumors and a worse prognosis. CSA was also inversely correlated with EOR in our data, suggesting that tumors with a larger surface area towards healthy brain tissue are more difficult to safely resect. Furthermore, the Kaplan-Meier curves in Fig. 3 suggest that the shape of the tumors had greater impact on the survival of patients who underwent subtotal resection compared to those who underwent gross total resection, and thus the gross total resection appears to be especially important in patients with irregular tumors. However, causality cannot be established from the current explorative study, and more research on the topic is required to determine an underlying mechanism for these findings.

The observed association between glioblastoma shape complexity and MGMT promoter methylation is also interesting and could be of relevance to the field of radiogenomics. A systematic review and meta-analysis found that tumors with methylated MGMT promoter are likely to show more edema, higher apparent diffusion coefficient, and lower perfusion [20]. A more recent review from 2022 showed that gliomas with methylated MGMT promoter are typically found in the temporal lobe of the left hemisphere and display ring contrast-enhancement, more central necrosis, higher minimum apparent diffusion coefficient, and higher relative cerebral blood flow [9]. Although surface area of the tumor is rarely mentioned in the literature, one study suggested that location and shape of the tumor were less predictive of MGMT promoter methylation status, as compared to textural characteristics [18]. Evidently, there is no obvious radiological phenotype related to MGMT promoter methylation in glioblastomas, and both methods and results of previous studies varied considerably. Nevertheless, the current study suggests that tumoral shape may be associated with MGMT promoter methylation, and thus it could be of interest for future studies.

This is to our knowledge the first study to assess contact surface area as a prognostic factor in glioblastoma patients. Strengths of this study include its rather large sample size, manually validated segmentations, and population-based patient selection. The greatest limitation is the retrospective approach without control of the data collection and follow-up, making it difficult to perform causal inferences. Regarding the statistical analyses, the presented p-values were not adjusted for multiple significance testing, which increases the risk of type I errors. However, as this study was performed in an exploratory setting given the scarce literature on this topic, we found it more important to avoid a large risk of type II errors than to minimize the risk of type I errors.

## Conclusion

Pretreatment tumor shape, measured as contact surface area and sphericity index, appears to be an independent prognostic factor in glioblastoma patients. Contact surface area may also be linked to MGMT promoter methylation status and extent of surgical resection.

## Data Availability

Data availabilityThe datasets presented in this article are not readily available because of restricted access by General Data Protection Regulation (GDPR). Requests to access the datasets should be directed to Claes.Johnstad@ntnu.no.

## Abbreviations

AIC: Akaike’s information criterion
A/V: area-to-volume ratio
C-index: Harrell’s concordance index
CSA: contact surface area
EOR: extent of resection
GTR: gross total resection
HR: hazard ratio
IDH: isocitrate dehydrogenase
KPS: Karnofsky performance status
MGMT: O6-methylguanine-DNA methyltransferase
MRI: magnetic resonance imaging
REK: Regional Committee for Medical and Health Research Ethics
SI: sphericity index
STR: subtotal resection
TSA: tumor surface area
WHO: World Health Organization
